# Etiological risk factors for infection in the postoperative period of cardiovascular surgery, integrative review

**DOI:** 10.15649/cuidarte.4607

**Published:** 2026-04-29

**Authors:** Reinaldo Gutiérrez Barreiro, Juan Sebastián Renza Molina, Paula Yisseth Cortes Motta, Juan Pablo Tavera Sánchez, María Camila Ortiz Zabaleta

**Affiliations:** 1 Universidad Surcolombiana, Programa de Enfermería, Neiva, Colombia. E-mail: reinaldo.gutierrez@usco.edu.co Universidad Surcolombiana Neiva Colombia reinaldo.gutierrez@usco.edu.co; 2 Universidad Surcolombiana, Programa de Enfermería, Neiva, Colombia. E-mail: u20211196530@usco.edu.co Universidad Surcolombiana Neiva Colombia u20211196530@usco.edu.co; 3 Universidad Surcolombiana, Programa de Enfermería, Neiva, Colombia. E-mail: paulitacortes2018@gmail.com Universidad Surcolombiana Neiva Colombia paulitacortes2018@gmail.com; 4 Universidad Surcolombiana, Programa de Enfermería, Neiva, Colombia. E-mail: u20201185948@usco.edu.co Universidad Surcolombiana Neiva Colombia u20201185948@usco.edu.co; 5 Universidad Surcolombiana, Programa de Enfermería, Neiva, Colombia. E-mail: u20221202962@usco.edu.co Universidad Surcolombiana Neiva Colombia u20221202962@usco.edu.co

**Keywords:** Infection, Risk Factors, Nursing Diagnosis, Cardiac Surgery, Intensive Care Unit, Infección, Factores de Riesgo, Diagnóstico de Enfermería, Cirugía Cardiaca, Unidad de Cuidados Intensivos, Infecção, Fatores de Risco, Diagnóstico de Enfermagem, Cirurgia Cardíaca, Unidade de Terapia Intensiva

## Abstract

**Introduction:**

Recognizing the elements related to the risk for infection during the postoperative period of cardiovascular surgery is essential for guiding effective strategies and strengthening clinical judgment.

**Objective:**

To identify the etiological factors associated with the nursing diagnosis "risk for infection" during the postoperative period of cardiovascular surgery in the intensive care unit from the scientific literature.

**Materials and Methods:**

An integrative literature review based on the methodology of Whittemore and Knafl. The search was conducted between August and September 2024 in the Scopus, ScienceDirect, PubMed, and CINAHL databases. The controlled descriptors used were Risk Factors, Risk, Infection, Nursing Diagnosis, Cardiovascular Surgical Procedures, and Intensive Care Units. The uncontrolled terms used were Risk of Infection, Cardiac Surgery, and Cardiovascular Surgery in English, Spanish, and Portuguese. After quality assessment, 25 articles met the criteria for data extraction.

**Results:**

36 etiological factors associated with the diagnosis "risk for infection" in the postoperative period of cardiovascular surgery were identified; of these, 14 were already described in NANDA-I, and 22 new factors are not included in this taxonomy.

**Discussion:**

The elements related to the reviewed diagnosis are grouped into three categories: patient antecedent factors, perioperative factors, and factors associated with the postoperative period in the ICU.

**Conclusion:**

The identification of new etiological factors associated with the diagnosis "risk for infection," specific to the target population, contributes to an understanding of the causality of this diagnosis and to determining nursing interventions based on the etiological factors identified during diagnostic reasoning.

## Resumen

**Introducción:**

Reconocer los elementos relacionados al riesgo de infección durante el posoperatorio de cirugía cardiovascular, resulta esencial para orientar estrategias eficaces y fortalecer el juicio clínico.

**Objetivo:**

Identificar en la literatura científica los factores etiológicos del diagnóstico de enfermería Riesgo de Infección durante el postoperatorio de cirugía cardiovascular en la unidad de cuidados intensivos.

**Materiales y métodos:**

Revisión integrativa de literatura basada en la metodología de Whittemore y Knafl. La búsqueda fue realizada entre agosto y septiembre del 2024 en las bases Scopus, ScienceDirect, PubMed y CINAHL, Se utilizaron los descriptores controlados: Factores de Riesgo, Riesgo, Infección, Diagnostico de Enfermería, Procedimientos Quirúrgicos Cardiovasculares y Unidad de Cuidados Intensivos. Y los términos no controlados Riesgo de Infección, Cirugía Cardiaca y Cirugía Cardiovascular en inglés, español y portugués. Después de verificar la calidad, 25 artículos cumplían los criterios para la extracción de la información.

**Resultados:**

Se identificaron 36 factores etiológicos del "riesgo de infección" en el posoperatorio de cirugía cardiovascular, de estos 14 ya estaban descritos en la NANDA-I y 22 nuevos no están incluidos en esta taxonomía.

**Discusión:**

Los elementos relacionados al diagnóstico revisado se agrupan en tres categorías: factores antecedentes del paciente, perioperatorios y asociados al postoperatorio en la UCI.

**Conclusiones:**

La identificación de nuevos factores etiológicos del diagnóstico Riesgo de Infección específicos de la población objeto, representa un aporte en la comprensión de la causalidad de este diagnóstico y en la determinación de las intervenciones de enfermería basadas en los factores etiológicos identificados durante el razonamiento diagnóstico.

## Resumo

**Introdução:**

Reconhecer os elementos relacionados ao risco de infecção no pós-operatório de cirurgia cardiovascular é essencial para orientar estratégias eficazes e fortalecer o julgamento clínico.

**Objetivo:**

Identificar na literatura científica os fatores etiológicos para o diagnóstico de enfermagem risco de infecção no pós-operatório de cirurgia cardiovascular em unidade de terapia intensiva.

**Materiais e métodos:**

Revisão integrativa da literatura, baseada na metodologia de Whittemore e Knafl. A busca foi realizada entre agosto e setembro de 2024 nas bases de dados Scopus, Science Direct, PubMed e Cinahl. Os descritores controlados utilizados foram: Fatores de Risco, Risco, Infecção, Diagnóstico de Enfermagem, Procedimentos Cirúrgicos Cardiovasculares e Unidade de Terapia Intensiva. Os termos não controlados utilizados foram Risco de Infecção, Cirurgia Cardíaca e Cirurgia Cardiovascular, nos idiomas inglês, espanhol e português. Após o controle de qualidade, 25 artigos atenderam aos critérios para extração dos dados.

**Resultados:**

Foram identificados 36 fatores etiológicos para "risco de infecção" no pós-operatório de cirurgia cardiovascular. Destes, 14 já estavam descritos na NANDA-I e 22 novos não constam nesta taxonomia.

**Discussão:**

Os elementos relacionados ao diagnóstico revisado são agrupados em três categorias: histórico do paciente, fatores perioperatórios e fatores associados ao pós-operatório em UTI.

**Conclusões:**

A identificação de novos fatores etiológicos para o diagnóstico "risco de infecção" específicos para a população-alvo representa uma contribuição para a compreensão da causalidade deste diagnóstico e para a determinação de intervenções de enfermagem com base nos fatores etiológicos identificados durante o raciocínio diagnóstico.

## Introduction

According to the World Health Organization (WHO), cardiovascular diseases (CVDs) are currently the leading cause of morbidity and mortality worldwide, with approximately 19.8 million people dying from this cause in 2022^1^. In Colombia, according to the National Administrative Department of Statistics (DANE), CVDs were one of the leading causes of death during 2022, with ischemic heart disease being the principal cause, accounting for 96.57 deaths per 100,000 inhabitants (41,783 cases)^2^. Thus, it is evident that cardiovascular diseases represent a significant public health problem due to their negative impact on the population's health, as well as the substantial demand for resources they place on health systems^3^. Those who suffer from CVDs require continuous and comprehensive care and, depending on the severity of the cardiac condition, may undergo surgical procedures such as cardiovascular surgery (CVS)^4^,^5^.

CVS procedures are complex and require specific care during the pre-, intra-, and postoperative stages. These procedures aim to contribute to the restoration of physiological balance with minimal complications and the highest quality of care. Therefore, this recovery process needs to take place in the Intensive Care Unit (ICU)^6^-^9^.

Infectious processes during the postoperative (POP) period of CVS can be related to the severity of the intervention. As a major surgical procedure, it entails a particularly high risk of infection, with incidence rates ranging from 3.5% to 26.8%^10^. Furthermore, this risk is also associated with pre-existing cardiac and non-cardiac comorbidities prior to the surgical procedure, which increase the likelihood of developing an infectious process. Likewise, it must be considered that patients undergoing this type of surgery require invasive devices and procedures, which, according to scientific evidence, are highly prone to colonization by pathogenic agents^11^,^12^,^13^.

Therefore, based on the preceding statements, the need for early identification of infection risk factors in patients is evident. For this purpose, nursing professionals have the nursing diagnosis "risk for infection," which is included in the North American Nursing Diagnosis Association International (NANDA-I) classification^14^. This tool guides and facilitates nursing professionals' clinical and diagnostic inference to identify patients susceptible to infections. Thus, this diagnosis can guide the nursing team' interventions, developing activities to mitigate these infectious processes^15^,^16^.

However, in the available scientific evidence, no study has been reported that investigates the causal elements of the diagnosis "risk for infection" among patients undergoing CVS during postoperative recovery in the ICU. Due to the situation described above, the present integrative review aimed to identify the etiological factors (EFs) associated with the nursing diagnosis "risk for infection" in postoperative cardiovascular surgery (POP CVS) patients admitted to the ICU, based on the available scientific evidence.

## Materials and Methods

An integrative literature review was conducted following the method proposed by Whittemore and Knafl, which facilitated the synthesis of knowledge found in the literature, along with an analysis and quality appraisal of primary and secondary research articles available in the evidence base. This model comprises five stages, enabling a critical review of the subject matter to address the research question^17^.

**Methodology**

The steps of the methodology proposed by Whittemore and Knafl^17^ comprise five stages for the correct execution and presentation of the results of an integrative literature review: 1) problem identification, 2) literature search, 3) data evaluation, 4) data analysis, and 5) presentation of the review findings.

**Search methods**

This review was guided by the central question: What etiological factors are associated with the nursing diagnosis "risk for infection" in POP CVS patients during their stay in the intensive care unit? The search strategy included the following keywords: Risk Factors, Nursing Diagnosis, risk, Infection, Hospital Infection, cardiac surgery. The information search strategy used different combinations of controlled descriptors (MeSH terms): "nursing diagnosis," "risk factors," "risk," "infection," "cardiovascular surgical procedures," and "intensive care units." The uncontrolled terms "cardiac surgery," "thoracic surgery," and "cardiovascular surgery" were also used and were combined using the Boolean operators "AND" and "OR." Table 1.

**Table 1:** Description of the search strategy in relation to databases and descriptors or terms used

Database	Search strategy
PubMed Scopus ScienceDirect CINHAL	Cardiac surgery AND risk factors AND infection AND intensive care unit
	Risk factor AND infections AND patient cardiovascular surgery AND intensive care unit

**Inclusion and exclusion criteria**

The inclusion criteria used for this review comprised articles in which the target population consisted of POP CVS patients admitted to the ICU who were at high risk of developing an infectious process. Additionally, articles published in English, Spanish, or Portuguese were selected, including studies published between 2014 and 2024. Furthermore, studies whose primary theme focused on the identification of EFs among these patients were selected. For this reason, quantitative studies employing any methodological design were included.

The exclusion criteria were as follows: manuscripts classified as gray literature, opinion articles, letters to the editor, information from academic events such as conference presentations, among others; and any article that obtained a final score lower than 50% on the application of the Crowe Critical Appraisal Tool (CCAT). The CCAT assesses the methodological quality of qualitative and quantitative scientific articles across eight categories: preliminary information, introduction, design, sampling, data collection, ethical considerations, results, and finally, discussion and conclusion^18^.

To ensure rigor in article selection and minimize potential biases, at least two reviewers independently evaluated all studies. Decisions regarding inclusion or exclusion of studies were made based on the initial screening of titles and abstracts. In cases of disagreement between the reviewers, discrepancies were resolved by a third evaluator.

**Data evaluation**

Upon completing the identification and extraction of information, the obtained data needed to be adequately ordered, coded, and classified^15^. An Excel matrix was created to record the data from each selected study, including database source, journal name, authors, article title, publication year, country where the study was conducted, country of publication, research approach, study design, population and sample, and main results. All collected data are available for open access and consultation in Mendeley^19^.

**Data analysis**

A critical reading of each selected article was performed by at least two reviewers. Subsequently, the findings of each study were discussed with the rest of the research team to ensure rigor in data identification and analysis. The extracted data were then systematized to develop the results. In this stage, the studies were first characterized based on the reported data and interpreted as possible EFs associated with the diagnosis "risk for infection" in POP CVS patients admitted to the ICU. Following Whittemore and Knafl's recommendations for correct data analysis, this process is divided into four steps: data reduction, data display, data comparison, and drawing conclusions^17^.

**Ethical considerations**

In developing this literature review, ethical principles of scientific research were upheld, ensuring integrity in data management, transparency in the analysis process, and appropriate acknowledgment of the contributions of all cited authors, in accordance with Law 1915 of 2018^20^. Since no procedures were performed on living beings, this study was considered to pose no risks and complied with the regulations of the Ministry of Health of Colombia^21^.

## Results

**Search results**

The initial search yielded a total of 1,977 articles, all in English, Spanish, or Portuguese. The search returned 632 records from PubMed, 438 from Scopus, 805 from ScienceDirect, and 102 from CINHAL. After applying the inclusion and exclusion criteria, a sample of 25 articles was selected for the development of this review.

**Descriptive results**

**Figure 1:**
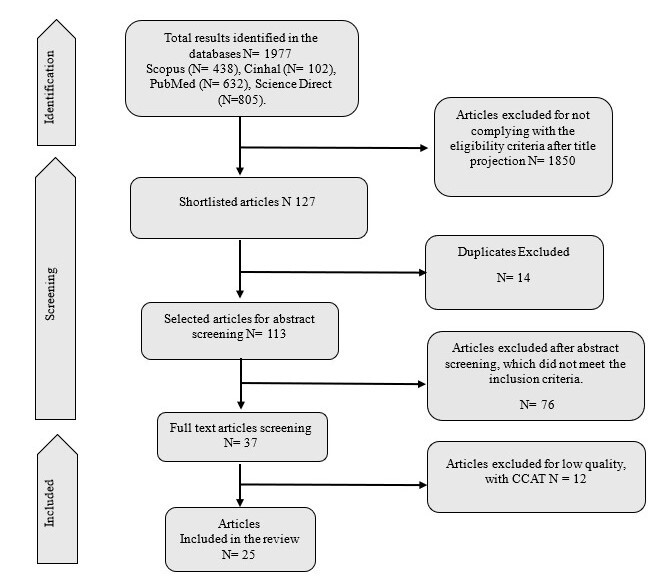
PRISMA flowchart for identification, selection, and inclusion of studies describing etiological factors associated with the diagnosis "risk for infection" in postoperative cardiovascular surgery patients in the ICU

*Note: CCAT: Crowe Critical Appraisal Tool.*

Of the 1,977 empirical and theoretical publications identified, 25 articles were included in this review. The selection process is illustrated in the PRISMA flow diagram, shown in Figure 1.

As observed in the flowchart, 25 articles that met the inclusion criteria were included in this review. From these articles, information reported in the literature on causal elements that increase the risk of infectious processes in the target study population was extracted, thereby addressing the guiding question of this research. It is noteworthy that none of the studies directly investigated these factors as EFs associated with the diagnosis "risk for infection." However, articles were found that reported different causal elements that can increase the risk of infection in this clinical context. Consequently, it was possible to interpret these elements as EFs associated with the diagnosis "risk for infection" in CVS patients in the ICU. Table 2 presents information on the included studies and the EFs identified.

**Table 2:** Characteristics of the articles included in the review and etiological factors associated with the diagnosis "risk for infection" in postoperative cardiovascular surgery patients.

Database	Authors and year	Study design	Sample size	Etiological factors
PubMed	Conoscenti et al. 2023^22^.	Historical cohort, single-center	3,609 cardiac surgery patients	• Diabetes mellitus • Renal insufficiency • Dialysis requirement • Previous CVS • Previous myocardial infarction • Overweight/obesity • Prolonged mechanical ventilation
	Wang et al. 2022^23^.	Retrospective observational	61 patients who developed ventilator-associated pneumonia	• Renal insufficiency • Prolonged extracorporeal circulation time • Nasogastric tube use
	Jiang et al. 2018^24^.	Retrospective observational	1,606 patient records	• Prolonged ICU stay • Prolonged surgical procedure duration • Intraoperative complication (stroke) • Preoperative hospitalization
	Alghamdi et al. 2022^25^.	Retrospective observational	2,366 patients	• Diabetes mellitus • Arterial hypertension • Smoking • Renal insufficiency • Surgical reintervention
	Cotogni et al. 2017^26^.	Prospective cohort	1,020 patients	• Failures in antibiotic prophylaxis administration
ScienceDirect	Li et al. 2019^27^.	Retrospective cohort	1,216 patients	• Previous myocardial infarction • Chronic infection • Renal insufficiency • Chronic obstructive pulmonary disease (COPD)
	Cutrell et al 2016^28^.	Case-control	39 DSWI cases and 117 controls (total 1894 surgeries)	• Requirement for multiple blood component transfusions • Chronic infection
	Tronstad et al. 2024^29^.	Quasi-experimental	68 patients	• Sleep deprivation
	Hughes et al. 2021^30^.	Case-control	52 cases and 104 controls	• Failures in antibiotic prophylaxis administration
	Andrioli et al. 2016^31^.	Quasi-experimental	330 patients	• Urinary catheterization
	Nešpor et al. 2015^32^.	Retrospective observational	9,110 patients	• Overweight/obesity • COPD • Smoking • Prolonged ICU stay • Orotracheal reintubation • Requirement for inotropic support (multiple, prolonged, higher dose)
	Del Val et al. 2022^33^.	Multicentric retrospective observational	604 patients	• Hemorrhage
Scopus	Ren et al. 2023^34^.	Retrospective observational	409 clinical records	• Surgical reintervention • Prolonged ICU stay • Failures in antibiotic prophylaxis administration
	Spagnolello et al. 2022^35^	Retrospective observational	611 patients	• Prolonged ICU stay • Prolonged extracorporeal circulation time
	de la Varga-Martínez et al. 2021^36^.	Prospective observational	1,097 patients	• Prolonged extracorporeal circulation time • Prolonged aortic clamping time • Prolonged use of blood catheters • Urinary catheterization • Prolonged mechanical ventilation • Prolonged ICU stay • Multiple cardiac surgical procedures • Requirement for emergency cardiac surgical procedure
	Wang et al. 2021^37^.	Retrospective cohort	322 patients	• Prolonged extracorporeal circulation time
	Liu et al. 2021^38^.	Retrospective observational	1360 patients	• Prolonged surgical procedure duration • Prolonged mechanical ventilation • Orotracheal reintubation • Tracheostomy
	Giacobbe et al. 2020^39^.	Case-control	222 patients (74 cases and 148 controls)	• Prolonged ICU stay • Failures in antibiotic prophylaxis administration • Heart failure
	McClure et al. 2019^40^.	Multicenter randomized controlled trial	7,507 patients	• Prolonged extracorporeal circulation time • Requirement for multiple blood component transfusions • Persistent hyperglycemia • Diabetes mellitus • Overweight/obesity
	Vicente-Martínez, R et al. 2019^41^.	Prospective observational	669 patients	• Prolonged ICU stay • Requirement for multiple blood component transfusions
	Järvelä et al. 2018^42^.	Prospective cohort	1,356 patients	• Persistent hyperglycemia
	Vondran et al. 2018^43^.	Retrospective cohort	41,466 patients	• Preoperative ventilation requirement • Requirement for multiple blood component transfusions • Prolonged extracorporeal circulation time • Prolonged aortic clamping time
CINAHL	Li et al. 2022^44^.	Case-control	503 patients	• Advanced age • Prolonged mechanical ventilation • Postoperative extracorporeal membrane oxygenation (ECMO) requirement
	Brunet, et al. 2020^45^.	Retrospective cohort	182 patients	• Overweight/obesity • Anemia • Chronic infection • Requirement for multiple blood component transfusions • Prolonged ICU stay
	Liu et al. 2016^46^.	Retrospective observational	2,108 patients	• Diabetes mellitus

CVS: cardiovascular surgery; ICU: Intensive Care Unit; COPD: Chronic obstructive pulmonary disease; ECMO: extracorporeal membrane oxygenation.

**Table 3:** Frequency of etiological factors associated with the diagnosis "risk for infection" in cardiovascular postoperative patients in the ICU found in the review

Type of etiological factor	Etiological factor	No. of articles describing the factor	Present in NANDA-I	Not present in NANDA-I
Clinical factors and comorbidities	Diabetes mellitus (Chronic disease)	4	^22^,^25^,^40^,^46^	
	Renal insufficiency (Chronic disease)	4	^22^,^23^,^25^,^27^	
	Overweight/obesity	3	^22^,^32^,^45^	
	Chronic infection (Chronic disease)	3	^27^,^28^,^45^	
	Smoking	2	^25^,^32^		
	Previous myocardial infarction	2		^22^,^27^
	COPD (Chronic disease)	1	^32^	
	Anemia	1	^45^	
	Arterial hypertension (Chronic disease)	1	^25^	
	Advanced age	1		^44^
	Previous CVS	1		^22^
	Dialysis requirement (Invasive procedure)	1	^22^	
	Heart failure (Chronic disease)	1	^39^	
	Preoperative hospitalization	1		^24^
	Preoperative ventilation requirement	1		^43^
Perioperative factors	Prolonged extracorporeal circulation time	6		^23^,^35^,^36^,^37^,^40^,^43^
	Failures in antibiotic prophylaxis administration	4		^26^,^30^,^34^,^39^
	Prolonged aortic clamping time	2		^36^,^43^
	Prolonged surgical procedure duration	2		^24^,^38^
	Surgical reintervention	2		^25^,^34^
	Intraoperative complication (stroke)	1		^24^
	Hemorrhage	1		^33^
	Requirement for emergency cardiac surgical procedure	1		^36^
	Multiple cardiac surgical procedures	1		^36^
ICU environment and recovery factors	Prolonged ICU stay	8		^24^,^32^,^34^,^35^,^36^,^39^,^41^,^46^
	Requirement for multiple blood component transfusions	5		^28^,^40^,^41^,^43^,^45^
	Prolonged mechanical ventilation	4		^22^,^36^,^38^,^44^
	Urinary catheterization (Invasive procedure)	2	^31^,^36^	
	Orotracheal reintubation	2		^32^,^38^
	Persistent hyperglycemia	2		^40^,^42^
	Nasogastric tube use (Invasive procedure)	1	^23^	
	Sleep deprivation	1		^29^
	Tracheostomy requirement (Invasive procedure)	1	^38^	
	Prolonged use of blood catheters (Invasive procedure)	1	^36^	
	Requirement for inotropic support (multiple, prolonged, higher dose)	1		^32^
	Postoperative ECMO requirement	1		^44^

CVS: cardiovascular surgery; ICU: Intensive Care Unit; COPD: Chronic obstructive pulmonary disease; ECMO: extracorporeal membrane oxygenation.

More than 50% of the included articles were analytical (cohort and case-control) and interventional studies, as well as retrospective observational research. This methodological heterogeneity strengthens the level of evidence for the causality of the reviewed diagnosis. Likewise, the sample sizes, which comprised a considerable number of participants in these studies, are noteworthy, reinforcing the external validity of the analyzed findings. Regarding the causal elements, the diverse nature of the EFs associated with the diagnosis "risk for infection" in patients during POP recovery in the ICU following CVS was evident. These factors ranged from pre-existing clinical conditions, perioperative factors, procedural factors, and factors specific to the intensive care environment, reflecting the complexity of the infectious process in this population. The classification of the analyzed EFs of the nursing diagnosis by different categories is presented in Table 3.

A total of 36 EFs associated with the diagnosis "risk for infection" among patients undergoing CVS were found in the consulted scientific evidence. Of these, more than 70% are not included in the current NANDA-I taxonomy. Regarding the types of EFs, a multifactorial nature was found, and they were classified into preoperative conditions (referring to 15 clinical antecedents and patient comorbidities), characteristics derived from the complexity of the procedure (corresponding to 9 perioperative factors), and aspects related to intensive therapeutic support (specifically, 12 ICU environment and recovery factors). Among these causal elements, those linked to postoperative management and prolonged life-support—such as prolonged ICU stay and extended mechanical ventilation—and those related to procedural complexity—such as the duration of extracorporeal circulation—were the most frequently reported in the reviewed literature.

## Discussion

**EFs associated with the diagnosis "risk for infection" in the cardiac postoperative period in the ICU related to patients’ clinical antecedents and comorbidities**

This review compiled 15 EFs specific to patients' clinical conditions and pathological histories. Among this group of causal elements, overweight and obesity stood out, which, in turn, were related to various comorbidities, such as diabetes mellitus, renal disease, and heart failure. This factor increases the risk of bacterial colonization in surgical wounds. Patients with diabetes and obesity (BMI >30) have a higher probability of postoperative infections, which may be exacerbated by decreased blood perfusion and associated vascular complications^43^,^45^. These findings align with those of Andrade et al. ^47^ in 2019, who reported that overweight patients and patients with obesity undergoing CVS were twice as likely to develop postoperative infections, primarily at the surgical site.

Furthermore, advanced age is also a critical EF because, as patients age, the immune system becomes less effective, diminishing its capacity to combat infections^44^. Similarly, renal insufficiency is related to comorbidities that affect the immune response. Riveros et al.^48^, monitoring a surgical cohort, reported that infectious complications were more common as the stage of renal insufficiency increased. Likewise, dialysis requirements, both peritoneal and hemodialysis, increase infection risk due to prolonged use of invasive devices, which raises the probability of handling errors that, in turn, can lead to serious infections^49^,^50^. Additionally, diabetes mellitus stood out as a causal element of this diagnosis due to its strong association with infection susceptibility related to metabolic alterations, pH imbalance, and hyperglycemia, which favor microbial growth. This situation is further aggravated by macro- and microvascular complications inherent to this disease, which affect wound healing and the immunological response^46^. These findings are consistent with those reported by Moorthy et al.^51^ who concluded that diabetes is associated with a greater risk of renal dysfunction, hyperglycemia, and infection following cardiac surgery.

On the other hand, smoking and its eventual pulmonary damage^25^,^32^, and the presence of COPD were EFs identified that amplify the risk of infection during the cardiac postoperative period, as patients with these respiratory conditions require longer periods of ventilatory support, making them susceptible to ventilator-associated pneumonia^32^. This information aligns with the results of Zhao et al.^52^, who found that COPD is associated with a higher risk of respiratory failure, renal insufficiency, pneumonia, stroke, and wound infection following coronary artery bypass graft surgery. Moreover, preoperative anemia was also identified as a relevant EF, as it decreases oxygen transport to tissues and impairs immunological function^45^. Similarly, patients with chronic infections (e.g., respiratory, urinary, cutaneous, or endovascular) may harbor latent pathogens in tissues for long periods and manifest clinically their proliferation in response to somatic stress produced by a surgical procedure and postoperative immunodepression. Therefore, these infections can reactivate or disseminate, causing bacteremia, sepsis, or surgical site infections^27^,^28^,^45^.

Other patient-specific EFs included a history of acute myocardial infarction^22^,^27^, arterial hypertension^25^, and heart failure^39^, all of which contribute to a vulnerable state of the patient as hemodynamic instability increases the risk of infection in this clinical context by compromising tissue perfusion and delaying healing, thereby favoring microbial colonization^25^,^53^. Finally, previous CVS, preoperative hospital stay, and the need for preoperative mechanical ventilation were EFs that increased the risk of infection. These conditions are related to greater clinical compromise—and thus a state of biological vulnerability— extended exposure to the hospital environment, and invasive procedures, conditions that collectively create a favorable scenario for the development of postoperative infections in these patients^22^,^24^,^43^.

**EFs associated with the diagnosis "risk for infection" in the cardiac postoperative period in the ICU related to perioperative aspects**

Among this set of 9 factors, failures in the administration of antibiotic prophylaxis stand out. Errors in prescribing and administering this therapy eliminate one of the primary preventive strategies against infections in these types of procedures. This situation facilitates bacterial colonization and pathogen dissemination in an unstable organism due to the hemodynamic shock characteristic of the postoperative CVS period, thereby breaching surgical safety protocols^26^,^30^,^35^.

Furthermore, cardiac surgical interventions are procedures that require extended operative times to correct the cardiovascular alterations present in the patients. Their complexity increases when different interventions are performed, such as the concomitant execution of multiple cardiac surgical procedures or undergoing emergency CVS. These circumstances, in turn, prolong the duration of the surgical procedure, the extracorporeal circulation time, and the aortic clamping time. The presence of these factors is related to patients' hemodynamic compromise and the magnitude of the intervention, which translates into a more severe systemic inflammatory response, hemodynamic and immunological alterations, and thus increased susceptibility to postoperative infections^35^,^36^,^40^,^43^. Although extracorporeal circulation and aortic clamping correspond to necessary life-support procedures during the surgical intervention^35^,^43^, prolonged duration of these procedures increases the probability of the analyzed diagnosis. For example, it has been reported that the infection risk increases significantly for every minute of extracorporeal circulation. Studies affirm that after 96 minutes, the risk of surgical site infections increases, and after 120 minutes, for other types of infections^35^,^36^. Finally, an aortic clamping time greater than 150 minutes is directly associated with bacterial colonization, as the procedure requires direct cardiac manipulation^36^.

Likewise, CVS are procedures with significant number of possible intraoperative complications. Among these potential negative consequences, surgical reintervention, intraoperative complication (stroke), and hemorrhage were identified as EFs associated with the diagnosis "risk for infection." These events demonstrate a more critical patient state and higher procedural complexity, both of which increase the risk of pathogen exposure and compromise the patient's immunological defense mechanisms. Thus, reopening surgical incisions, repeated invasive interventions, and greater tissue damage all favor microbial colonization and infection development. Intraoperative adverse events, specifically stroke, prolong surgical duration and ICU stay, increase the use of invasive devices, and the risk of respiratory infections in the POP. Hemorrhage, in turn, induces immunosuppression, delays healing, and requires additional interventions, increasing the possibility of contamination^24^,^25^,^33^,^34^.

**EFs associated with the diagnosis "risk for infection" in the cardiac postoperative period in the ICU, related to the ICU environment and recovery**

Patients undergoing CVS must be immediately transferred to the ICU, as they present significant physiological alterations during the POP of this highly complex intervention. These patients usually require ventilatory and vasoactive support, as well as close monitoring to ensure hemodynamic, respiratory, and metabolic stability and tissue perfusion, allowing timely detection and treatment of complications^54^. However, intensive care implies exposure to different risks of microbial colonization. Prolonged ICU stay is an EF that impacts patient mortality, whether due to complications of the underlying disease and surgery or due to hospital-acquired infections^24^. This risk is directly related to the use of invasive devices, which, although fundamental for life support, can serve as potential entry routes for pathogens, especially when their use is extended^26^.

Another EF is prolonged mechanical ventilation, an essential procedure to ensure adequate oxygenation during surgery and the postoperative period. However, durations exceeding 30 hours have been identified as a significant risk factor in the development of respiratory infections, especially pneumonia^36^. Likewise, airway infections are associated with failed weaning from ventilatory support, requiring orotracheal reintubation, which increases the risk of pneumonia due to airway manipulation, the patient's clinical state, and prolonged dependence on respiratory support^32^,^38^. Similarly, a tracheostomy is often necessary in cases of altered oxygenation after CVS or a high risk of aspiration; however, this procedure can also favor the development of pneumonia^45^,^53^,^55^. This argument is supported by Reyes et al., in 2023, who concluded that tracheostomy can be associated with an increase in complications, most prominently infections, with incidence rates ranging from 5% to 40%^56^.

Furthermore, management failures and prolonged use of venous catheters, especially central lines, in critically ill patients are associated with bloodstream infections, which can lead to the development of potentially fatal bacteremia or sepsis^57^. Likewise, urinary catheterization management errors constitute an independent factor for the onset of urinary tract infections during the POP of CVS^31^,^36^. Similarly, feeding tubes are often used in intubated patients for enteral nutrition, medication administration, or gastric decompression. It is noteworthy that the nasogastric route has been associated with an increased risk of gram-negative bacterial infections in ICU patients, particularly those with invasive ventilatory support^58^.

In addition to the use of multiple invasive devices, it is important to consider that the immunological status of these patients is often compromised due to surgical stress, systemic inflammatory response, hemodilution, prolonged exposure to immunomodulatory medications, and the presence of surgical incisions—factors that make them especially vulnerable to infectious processes^10^. Likewise, EFs such as the requirement for multiple blood component transfusions have been related to a higher infection risk during intraoperative and POP phases of CVS. Although transfusions are essential for correcting hemodynamic instability, it is fundamental to consider the quantity, quality, and technique of the transfused blood products^28^,^35^. This aligns with the findings of Al-Harbi et al.^59^, who documented that patients receiving blood transfusions at any point during the intraoperative or postoperative period were 2.6 times more likely to develop an infection than those who did not.

The requirement for inotropic support (multiple, prolonged, or high-dose), as well as the use of ECMO in the postoperative period, are considered EFs associated with the diagnosis "risk for infection," as they are closely related to a more compromised hemodynamic state. These medications are typically administered to patients with severe ventricular dysfunction to maintain adequate tissue perfusion and stable cardiac output. However, persistent hypoperfusion, endothelial damage, and organ dysfunction associated with this condition create a physiological environment conducive to microbial colonization^32^. Similarly, the application of ECMO represents a highly specialized procedure reserved for patients with extreme hemodynamic instability or cardiopulmonary failure refractory to conventional treatments. ECMO implementation requires the insertion of large-bore central catheters and the continuous extraction and re-infusion of blood through an extracorporeal circuit, which increases the risk of contamination^44^. According to Biffi et al.^60^, ECMO therapy increases the prevalence of nosocomial infections by approximately 10% to 12%.

Persistent hyperglycemia is another EF observed in critically ill patients, including those undergoing CVS, and is a frequent metabolic alteration that occurs even in individuals without a prior diabetes diagnosis. Persistent hyperglycemia is primarily explained by the activation of the neuroendocrine stress response to surgery, which triggers an increased secretion of catecholamines, cortisol, glucagon, and growth hormone. These hormones stimulate hepatic gluconeogenesis and glycogenolysis. Persistent hyperglycemia has significant clinical consequences, as it is associated with neutrophil and macrophage dysfunction, impaired chemotaxis and phagocytosis, delayed wound healing, and compromised inflammatory response. These mechanisms diminish the immune system's capacity to eliminate pathogens^42^,^61^.

Finally, sleep deprivation induces a state of chronic physiological stress that elevates cortisol and other counterregulatory hormones, affecting epithelial barrier function and delaying tissue repair processes. These changes favor microbial colonization and reduce the organism's ability to contain or eliminate pathogens, thereby increasing susceptibility to respiratory and surgical site infections, and bacteremia^29^,^62^.

In summary, it is evident that the EFs associated with the diagnosis "risk for infection" in the POP of CVS patients, as reported in the scientific evidence, demonstrate great variability and confirm the multifactorial causality of this human response in the clinical context of patients admitted to the ICU following CVS. This finding provides insights into the occurrence of this diagnosis and guides nursing professionals in clinical settings to implement interventions aimed at reinforcing the prevention of infectious processes in this population. One limitation observed in this review was that none of the included studies addressed the risk of infection as a diagnosis. However, by using the rigorous and systematic methodological approach proposed by Whittemore and Knafl, as well as the quality assessment of the articles and the interpretation of causal elements as EFs of the reviewed diagnosis, it was possible to achieve the proposed objectives.

## Conclusions

This review identified 36 EFs associated with the nursing diagnosis "risk for infection," reinforcing its relevance as a fundamental component of nursing diagnosis taxonomy within the nursing process. Of these, 22 factors are not currently described as causal for this human response in the NANDA-I classification. It should be noted that the present review was based on studies reporting this problem in the cardiac POP period in the ICU, rather than in the general population.

From the analysis conducted, it is evident that the etiological relationship of this diagnosis, as presented in the NANDA-I taxonomy, is limited. This finding is relevant, as the selection of nursing interventions depends directly on the accurate identification of these factors during the diagnostic reasoning process. Therefore, the findings of this review broaden the reference framework and promote a more comprehensive approach to action that considers not only patient antecedents but also perioperative factors, the recovery process, and the specific ICU environment.

These insights favor the development of more effective nursing care plans and models, while also allowing nursing professionals to be better informed about the diverse causal mechanisms of infection in the context of CVS POP. Finally, the results of this review lay the foundation for future studies that delve deeper into the causal relationships among these EFs and support their inclusion in subsequent updates of the NANDA-I taxonomy to improve the causal structure of this diagnosis.

## References

[1] Organizacion Mundial de la Salud (OMS) (2024). Cardiovascular diseases (CVDs) [Internet].

[2] Departamento Administrativo Nacional de Estadística DANE Defunciones no Fetales 2022.

[3] Ramic-Catak  A, Mesihović-Dinarevic  S, Prnjavorac  B, Naser  N, Masic  I (2023). Public health dimensions of CVD prevention and control - global perspectives and current situation in the Federation of BiH. Mater Socio Med.

[4] Méndez García  JE, Salinas Martínez  RD, Zambrano Sangurima  MS, Tomalá Ruiz  RD (2022). Cirugía coronaria mínimamente invasiva. RECIMUNDO.

[5] Birger  M, Kaldjian  AS, Roth  GA, Moran  AE, Dieleman  JL, Bellows  BK (2021). Spending on cardiovascular disease and cardiovascular risk factors in the United States: 1996 to 2016. Circulation.

[6] Keeling-Johnson  K, Baker  D, Want  T, Tuazon  DM (2023). Immediate postoperative management of cardiac surgery patients. Methodist DeBakey Cardiovasc J.

[7] Jiménez Rivera  JJ, Llanos-Jorge  C, López Gude  MJ, Pérez Vela  JL (2021). Perioperative management in cardiovascular surgery. Medicina Intensiva.

[8] Pahwa  S, Bernabei  A, Schaff  H, Stulak  J, Greason  K, Pochettino  A (2021). Impact of postoperative complications after cardiac surgery on long-term survival. J Card Surg.

[9] Pokhrel  S, Gregory  A, Mellor  A (2021). Perioperative care in cardiac surgery. BJA Educ.

[10] Wang  Y, Ren  J, Yao  Z, Wang  W, Wang  S, Duan  J (2023). Clinical impact and risk factors of intensive care unit-acquired nosocomial infection: a propensity score-matching study from 2018 to 2020 in a teaching hospital in China. Infect Drug Resist.

[11] Schiefenhövel  F, Trauzeddel  RF, Sander  M, Heringlake  M, Groesdonk  HV, Grubitzsch  H (2021). High central venous pressure after cardiac surgery might depict hemodynamic deterioration associated with increased morbidity and mortality. J Clin Med.

[12] García Carranza  A, Caro Pizarro  V, Quirós Cárdenas  G, Monge Badilla  MJ, Arroyo Quirós  A (2020). Catéter venoso central y sus complicaciones. Revista Medicina Legal de Costa Rica.

[13] Simões  AMN, Vendramim  P, Pedreira  MLG (2022). Risk factors for peripheral intravenous catheter- related phlebitis in adult patients. Rev Esc Enferm USP.

[14] Herdman  TH, Kamitsuru  S, Lopes  CT, Editores (2021). Diagnósticos enfermeros: Definiciones y clasificación, 2021-2023.

[15] Todo Bom  LFP, Mata  ESF, Cunha  HMP, Marquês  MdCM, Dixe  MdA (2025). Effectiveness of nursing interventions on preventing the risk of infection in adult inpatients: protocol for a systematic review. Nurs Rep.

[16] Marques  C da C, Silva  BCO da, Barreto  VP, Feitoza  AR, Lira  ALB de C, Feijão  AR (2021). Accuracy of risk factors for nursing diagnosis risk of infection in people with AIDS. Rev Esc Enferm USP.

[17] Whittemore  R, Knafl  K (2005). The integrative review: updated methodology. J Adv Nurs.

[18] Crowe  M, Sheppard  L, Campbell  A (2012). Reliability analysis for a proposed critical appraisal tool demonstrated value for diverse research designs. J Clin Epidemiol.

[19] Gutiérrez-Barreiro  R, Renza-Molina  JS, Cortes-Motta  PY, Tavera-Sánchez  JP, Ortiz-Zabaleta  MC (2025). Data-Set Factores etiológicos del diagnóstico riesgo de infección en pacientes postoperatorios de cirugía cardiovascular. Mendeley Data V2.

[20] Congreso de Colombia (2018). Ley 1915 de 2018: por la cual se modifica la Ley 23 de 1982 y se establecen otras disposiciones en materia de derecho de autor.

[21] Ministerio de Salud de Colombia (1993). Resolución número 8430 de 1993: por la cual se establecen las normas científicas, técnicas y administrativas para la investigación en salud.

[22] Conoscenti  E, Enea  G, Deschepper  M, Huis In 't Veld  D, Campanella  M, Raffa  G (2024). Risk factors for surgical site infection following cardiac surgery in a region endemic for multidrug resistant organisms. Intensive Crit Care Nurs.

[23] Wang  M, Xu  X, Wu  S, Sun  H, Chang  Y, Li  M (2022). Risk factors for ventilator-associated pneumonia due to multi-drug resistant organisms after cardiac surgery in adults. BMC Cardiovasc Disord.

[24] Jiang  WL, Hu  XP, Hu  ZP, Tang  Z, Wu  HB, Chen  LH (2018). Morbidity and mortality of nosocomial infection after cardiovascular surgery: a report of 1606 cases. Curr Med Sci.

[25] Alghamdi  BA, Alharthi  RA, AlShaikh  BA, Alosaimi  MA, Alghamdi  AY, Yusnoraini  N (2022). Risk factors for post-cardiac surgery infections. Cureus.

[26] Cotogni  P, Barbero  C, Passera  R, Fossati  L, Olivero  G, Rinaldi  M (2017). Violation of prophylactic vancomycin administration timing is a potential risk factor for rate of surgical site infections in cardiac surgery patients: a prospective cohort study. BMC Cardiovasc Disord.

[27] Li  S, Tang  BY, Zhang  B, Wang  CP, Zhang  WB, Yang  S (2019). Analysis of risk factors and establishment of a risk prediction model for cardiothoracic surgical intensive care unit readmission after heart valve surgery in China: a single-center study. Heart Lung.

[28] Cutrell  JB, Barros  N, McBroom  M, Luby  J, Minhajuddin  A, Ring  WS (2016). Risk factors for deep sternal wound infection after cardiac surgery: influence of red blood cell transfusions and chronic infection. Am J Infect Control.

[29] Tronstad  O, Patterson  S, Zangerl  B, Flaws  D, Holdsworth  R, Irvine  L (2024). The introduction of a sound reduction bundle in the intensive care unit and its impact on sound levels and patients. Aust Crit Care.

[30] Hughes  A, Sullivan  SG, Marshall  C (2021). Factors associated with vanA VRE acquisition in cardiothoracic surgery patients during an acute outbreak. Infect Dis Health.

[31] Andrioli  ER, Furtado  GH, Medeiros  EA (2016). Catheter-associated urinary tract infection after cardiovascular surgery: impact of a multifaceted intervention. Am J Infect Control.

[32] Nešpor  D, Fabián  J, Němec  P (2015). A retrospective analysis of deep sternal wound infections after longitudinal median sternotomy. Cardiovascular Surgery. Cor et Vasa.

[33] Del Val  D, Abdel-Wahab  M, Mangner  N, Durand  E, Ihlemann  N, Urena  M (2022). Infective endocarditis caused by Staphylococcus aureus after transcatheter aortic valve replacement. Can J Cardiol.

[34] Ren  J, Duan  S, Wu  Y, Wen  M, Zhang  J, Liu  Y (2023). Multidrug-resistant bacterial infection in adult patients following cardiac surgery: clinical characteristics and risk factors. BMC Cardiovasc Disord.

[35]  Spagnolello  O,  Fabris  S, Portella  G, Raafat Shafig Saber  D, Giovanella  E, Badr Saad  M (2022). Rates and determinants of hospital-acquired infection among ICU patients undergoing cardiac surgery in developing countries: results from EMERGENCY'NGO's hospital in Sudan. Antibiotics.

[36] de la Varga-Martínez  O, Gómez-Sánchez  E, Muñoz  MF, Lorenzo  M, Gómez-Pesquera  E, Poves- Álvarez  R (2021). Impact of nosocomial infections on patient mortality following cardiac surgery. J Clin Anesth.

[37] Wang  J, Wang  L, Jia  M, Du  Z, Hou  X (2021). Extracorporeal membrane oxygenation-related nosocomial infection after cardiac surgery in adult patients. Braz J Cardiovasc Surg.

[38] Liu  Z, Zhang  X, Zhai  Q (2021). Clinical investigation of nosocomial infections in adult patients after cardiac surgery. Medicine.

[39] Giacobbe  DR, Salsano  A, Del Puente  F, Miette  A, Vena  A, Corcione  S (2020). Risk factors for candidemia after open heart surgery: results from a multicenter case-control study. Open Forum Infect Dis.

[40] McClure  GR, Belley-Cote  EP, Harlock  J, Lamy  A, Stacey  M, Devereaux  PJ (2019). Steroids in cardiac surgery trial: a substudy of surgical site infections. Can J Anaesth.

[41] Vicente-Martínez  L, Vicente-Guillen  R, Calabuig  E, Escribá  F, Pajares  A, Argente  P (2019). Infección fúngica tras cirugía cardiaca. Nuestra experiencia. Rev Esp Anestesiol Reanim.

[42] Järvelä  KM, Khan  NK, Loisa  EL, Sutinen  JA, Laurikka  JO, Khan  JA (2018). Hyperglycemic episodes are associated with postoperative infections after cardiac surgery. Scand J Surg.

[43] Vondran  M, Schack  S, Garbade  J, Binner  C, Mende  M, Rastan  AJ (2018). Evaluation of risk factors for a fulminant Clostridium difficile infection after cardiac surgery: a single-center, retrospective cohort study. BMC Anesthesiol.

[44] Li  X, Wang  L, Li  C, Wang  X, Hao  X, Du  Z (2022). A nomogram to predict nosocomial infection in patients on venoarterial extracorporeal membrane oxygenation after cardiac surgery. Perfusion.

[45] Brunet  A, N'Guyen  Y, Lefebvre  A, Poncet  A, Robbins  A, Bajolet  O (2020). Obesity and preoperative anaemia as independent risk factors for sternal wound infection after coronary artery bypass graft surgery with pedicled (non-skeletonized) internal mammary arteries: the role of thoracic wall ischemia?. Vasc Health Risk Manag.

[46] Liu  Y, Han  J, Liu  T, Yang  Z, Jiang  H, Wang  H (2016). The effects of diabetes mellitus in patients undergoing off-pump coronary artery bypass grafting. Biomed Res Int.

[47] Andrade  LS de, Siliprandi  EMO, Karsburg  LL, Berlesi  FP, Carvalho  OL da F, Rosa  DS da (2019). “Bundle” de prevenção de sítio cirúrgico em cirurgia cardíaca. Arq Bras Cardiol.

[48] Riveros  C, Ranganathan  S, Shah  YB, Huang  E, Xu  J, Hsu  E (2024). Association of chronic kidney disease with postoperative outcomes: a national surgical quality improvement program (NSQIP) multi-specialty surgical cohort analysis. BMC Nephrol.

[49] Andreoli  MCC, Totoli  C (2020). Peritoneal dialysis. Rev Assoc Med Bras.

[50] Murdeshwar  HN, Agarwal  A, Anjum  F Hemodialysis. In: StatPearls. Treasure Island (FL): StatPearls Publishing.

[51] Moorthy  V, Liu  W, Chew  STH, Ti  LK (2019). Impact of diabetes on outcomes of cardiac surgery in a multiethnic Southeast Asian population. Diab Vasc Dis Res.

[52] Zhao  H, Li  L, Yang  G, Gong  J, Ye  L, Zhi  S (2019). Postoperative outcomes of patients with chronic obstructive pulmonary disease undergoing coronary artery bypass grafting surgery: a meta- analysis. Medicine.

[53]  Abukhodair  A, Alqarni  MS, Alzahrani  A, Bukhari  ZM, Kadi  A, Baabbad  FM (2023). Risk factors for postoperative infections in cardiac surgery patients: a retrospective study. Cureus.

[54] Gewarges  M, Cao  A, Alexopoulos  K, Al-Mandhari  M, Billia  F, Massarella  D (2025). Caring for two: management of the critically ill cardiac patient during pregnancy. JACC Adv.

[55] Chen  X, Yan  L, Zhao  S, Hu  X, Shao  G, Li  N (2025). Independent risk factors and outcomes for ventilator-associated pneumonia due to multidrug-resistant organisms after cardiac valvular surgery. Front Cardiovasc Med.

[56] Reyes Pulido  MM, Orozco Levi  M, Ramírez Sarmiento  AL, Nariño Gamboa  AJ, Fragozo Ibarra  AG (2022). Complicaciones en pacientes usuarios de traqueostomía en unidades de cuidados intensivos: scoping review. Rev Cuidarte.

[57] Silveira Borges  R, Estrin  M (2023). Incidence of infections in adult patients with central venous catheter: A systematic review. AG Salud.

[58] Maina  JW, Onyambu  FG, Kibet  PS, Musyoki  AM (2023). Multidrug-resistant Gram-negative bacterial infections and associated factors in a Kenyan intensive care unit: a cross-sectional study. Ann Clin Microbiol Antimicrob.

[59] Al-Harbi  SA, Alkhayal  N, Alsehali  A, Alshaya  S, bin Obaid  W, Althubaiti  A (2019). Impact of blood transfusion on major infection after isolated coronary artery bypass surgery: Incidence and risk factors. J Saudi Heart Assoc.

[60] Biffi  S, Di Bella  S, Scaravilli  V, Peri  AM, Grasselli  G, Alagna  L (2017). Infections during extracorporeal membrane oxygenation: epidemiology, risk factors, pathogenesis and prevention. Int J Antimicrob Agents.

[61] Vedantam  D, Poman  DS, Motwani  L, Asif  N, Patel  A, Anne  KK (2022). Stress-Induced Hyperglycemia: Consequences and Management. Cureus.

[62] Miranda-Ackerman  RC, Lira-Trujillo  M, Gollaz-Cervantez  AC, Cortés-Flores  AO, Zuloaga- Fernández del Valle  CJ, García-González  LA (2020). Associations between stressors and difficulty sleeping in critically ill patients admitted to the intensive care unit: a cohort study. BMC Health Serv Res.

